# Cerebral Blood Flow under Pressure: Investigating Cerebrovascular Compliance with Phase-contrast Magnetic Resonance Imaging during Induced Hypertension

**DOI:** 10.1097/ALN.0000000000005651

**Published:** 2025-07-11

**Authors:** Jonas Österlind, Johan Birnefeld, Anders Eklund, Magnus Hultin, Anders Wåhlin, Petter Holmlund, Laleh Zarrinkoob

**Affiliations:** 1 Department of Diagnostics and Intervention, Anesthesiology and Intensive Care, Umeå University, Umeå, Sweden.; 2 Department of Clinical Sciences, Neurosciences, Umeå University, Umeå, Sweden.; 3 Department of Diagnostics and Intervention, Biomedical Engineering and Radiation Physics, Department of Applied Physics and Electronics, Umeå University, Umeå, Sweden.; 4 Department of Diagnostics and Intervention, Anesthesiology and Intensive Care, Umeå University, Umeå, Sweden.; 5 Department of Diagnostics and Intervention, Umeå Centre for Functional Brain Imaging, Department of Applied Physics and Electronics, Umeå University, Umeå, Sweden.; 6 Department of Applied Physics and Electronics, Umeå University, Umeå, Sweden.; 7 Department of Diagnostics and Intervention, Anesthesiology and Intensive Care, Umeå University, Umeå, Sweden.

## Abstract

**Background:**

Induced hypertension is used clinically to increase cerebral blood flow (CBF) in conditions such as vasospasm after subarachnoid hemorrhage. However, increased blood pressure also raises pulsatile force. Cerebrovascular compliance plays a key role in buffering flow dynamics and protecting the microcirculation, but whether it adapts to elevated pressure remains unclear. This study assessed the response of compliant cerebral arteries to induced hypertension in healthy adults using phase-contrast magnetic resonance imaging (PCMRI) and two compliance models: a two-element Windkessel (compliance estimated using the Windkessel model, C_WK_) and a simplified model (compliance calculated as the ratio of pulsatile volume to pressure, C_VP_), representing the extremes of pulsatility transmission at the capillary level.

**Methods:**

Eighteen healthy adults (median age, 34 yr; nine women) underwent PCMRI at baseline and after increasing mean arterial pressure by 20% using norepinephrine infusion. PCMRI quantified CBF and cardiac output, while cerebrovascular resistance and systemic vascular resistance were derived. Flow waveforms were combined with blood pressure to assess C_WK_ and C_VP_ in CBF, ascending/descending aorta, and external carotid arteries, while corresponding regions of interest were used to calculate cross-sectional flow areas. Data are reported as median (interquartile range).

**Results:**

Norepinephrine increased cerebrovascular compliance significantly: C_WK_ by 110% (56 to 163%; *P* = 0.001) and C_VP_ by 11% (−2 to 26%; *P* = 0.018). C_WK_ increased in the external carotid artery by 12% (1 to 32%; *P* = 0.037) but did not change in the ascending or descending aorta. C_VP_ decreased in the descending aorta by 5% (−11 to 2%; *P* = 0.028), with no changes in the ascending aorta or external carotid artery. Cross-sectional area of cerebral arteries contributing to CBF decreased by 5% (−17 to −3%; *P* = 0.033), while the ascending and descending aorta areas increased by 7% (4 to 11%; *P* = 0.012) and 8% (6 to 11%; *P* < 0.001), respectively.

**Conclusions:**

Cerebral arteries enhanced their compliance during norepinephrine-induced hypertension, unlike systemic arteries, regardless of the assumed degree of pulsatility transmission.

## Abstract 2

Phase-contrast magnetic resonance imaging information was used in two models of cerebral blood flow and vascular compliance during induced hypertension. Induced hypertension resulted in an adaptive increase in cerebrovascular compliance, thus limiting excessive pulsatile flow in the cerebral microcirculation. This increase in arterial compliance was not seen in the systemic circulation.

## Editor’s Perspective

**What We Already Know about This Topic**

- Arterial vascular compliance dampens the downstream effects of pulsatile variations in arterial blood pressure and hence protects the cerebral microcirculatory flow

**What This Article Tells Us That Is New**

- Phase-contrast magnetic resonance imaging information was used in two models of cerebral blood flow and vascular compliance during induced hypertension
- Induced hypertension resulted in an adaptive increase in cerebrovascular compliance, thus limiting excessive pulsatile flow in the cerebral microcirculation
- This increase in arterial compliance was not seen in the systemic circulation

Induced hypertension is used in clinical practice to increase cerebral blood flow (CBF), for example, in cases of vasospasm after aneurysmal subarachnoid hemorrhage or during carotid endarterectomy.^1,2^ While the relationship between blood pressure and CBF is central to ensuring optimal oxygen delivery to the brain, the exact mechanisms remain unclear, despite being rooted in the principle of autoregulation as described by Lassen.^3^ Recent evidence highlights the significant role of cerebrovascular compliance in the relationship between blood pressure and CBF, offering a more comprehensive perspective on cerebrovascular dynamics.^4–6^ Vascular compliance attenuates abrupt blood pressure fluctuations by enabling arteries to stretch under pressure and return to their original shape without permanent deformation, effectively dampening the pulsatile flow.^7^ Understanding cerebrovascular compliance may, therefore, be crucial for a more comprehensive insight into the complex interplay between blood pressure and CBF.

We have recently shown using phase-contrast magnetic resonance imaging (PCMRI) that induced hypertension by increasing mean arterial pressure (MAP) by approximately 20% using norepinephrine in otherwise healthy individuals resulted in a reduction of CBF, cardiac output, and heart rate, along with an increase in cerebrovascular resistance (CVR) and pulse pressure (PP).^8^ Considering the increase in CVR and the reduction in heart rate, indicative of a protective cerebral autoregulatory response and baroreflex, respectively, elevating blood pressure beyond normal levels appears to be an undesirable strategy for enhancing CBF. Whether this holds true for the adaptive response of the compliant cerebral arteries remains to be investigated.

In recent years, substantial progress has been made in the study of cerebrovascular compliance, utilizing advanced imaging techniques and modeling strategies.^9,10^ One such approach uses compliance models that integrate blood pressure measurements with PCMRI.^11,12^ The feasibility of PCMRI for assessing CBF was demonstrated as early as the 1990s and has since been validated in various physiologic and clinical contexts, particularly within cardiology and neurology, including preoperative assessments before certain cardiac surgeries and interventions.^13–18^ It is also widely regarded as the accepted standard for noninvasive quantification of blood flow and its pulsatile characteristics.^16^ Building on this foundation, we extended PCMRI-based analysis to include vascular compliance modeling, aiming to characterize the physiologic response of the cerebral circulation during induced hypertension in awake, healthy individuals. We hypothesized that induced hypertension causes an adaptive increase in cerebrovascular compliance, acting to limit the transmission of flow pulsatility to the cerebral microcirculation.

## Materials and Methods

In a recent study, we investigated CBF changes during blood pressure alteration.^8^ Here we extend the analysis to study arterial compliance during induced hypertension. The study took place at Umeå University Hospital (Umeå, Sweden) between May 2021 and January 2022. Eighteen healthy, awake volunteers participated. CBF was measured, using PCMRI, at baseline and after increasing MAP by 20% using norepinephrine. All participants provided written informed consent. The Swedish Ethical Review Authority (Stockholm, Sweden) approved the study (approval No. 2020-05764), complying with the 1964 Declaration of Helsinki and its subsequent amendments.

### Study Population

Healthy individuals age 30 to 50 yr were recruited *via* hospital advertisements and social media. A physician (J.B.) assessed participants’ health with medical history, examinations, and electrocardiogram. Magnetic resonance imaging (MRI) screening excluded brain abnormalities. Exclusion criteria included cardiovascular or neurologic diseases, body mass index less than 18.5 or greater than 29.9, any pathology on electrocardiogram abnormalities, or contraindications to norepinephrine.

### Treatment Procedure

**Fig. 1.**
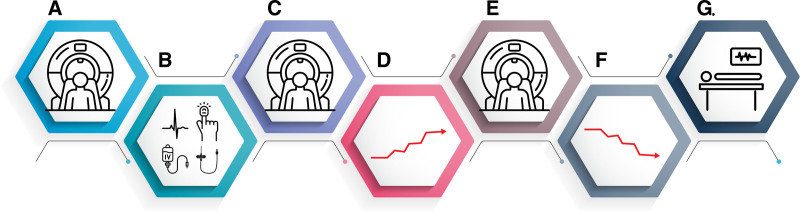
Schematic overview of the experimental setup and procedure. Step-by-step representation of the study protocol. (*A*) Structural magnetic resonance imaging (MRI) was performed and reviewed by a neuroradiologist. Participants with pathologic findings were excluded and referred for clinical follow-up, while those with normal imaging proceeded to the intervention MRI. (*B*) Medical history was updated, and participants were prepared for imaging. They were positioned on the MRI gurney, a radial arterial line was placed in the nondominant hand, and a venous catheter was inserted (preferably in the right antecubital vein). Participants were informed about common MRI-related sensations (*e.g.*, vibration, warmth, loud noises, emotional responses) and reminded they could communicate with staff at any time. Electrocardiogram and pulse oximeter monitoring were applied, and physiologic monitoring commenced. (*C*) Baseline MRI sequences were acquired, including phase-contrast MRI (PCMRI) at the level of the ascending and descending aorta, as well as at the C2–C3 level of the neck. (*D*) Norepinephrine infusion was initiated at 0.04 µg · kg^–1^ · min^–1^. The infusion rate was increased every 2 min by 0.01 to 0.04 µg · kg^–1^ · min^–1^, depending on the participant’s hemodynamic response, until the target mean arterial pressure (MAP) increase of +30% was reached (a +20% increase was accepted as sufficient). The infusion had no predetermined upper limit and was discontinued immediately if adverse effects occurred. After reaching target MAP, adjustments to the infusion rate were allowed to maintain the desired pressure. (*E*) MRI sequences were repeated during ongoing norepinephrine infusion, including PCMRI at the level of the ascending and descending aorta, as well as at the C2–C3 level of the neck. (*F*) The norepinephrine infusion was stopped, and the participant was monitored until blood pressure returned to baseline MAP. (*G*) The participant was transferred on the MRI gurney to the preparation room. Monitoring equipment and the arterial line were removed. The participant was then assisted to slowly sit up and rise from the gurney and remained under observation. Peripheral venous access was removed before the participant left the facility.

Figure 1 provides an overview of the experimental setup and procedure. Participants were not permitted nicotine, caffeine, alcohol, or exercise for 12 h before MRI investigation. Blood pressure and vital signs were monitored using Philips Expression MR400 (Philips Healthcare, The Netherlands) with a radial artery catheter, continuous electrocardiogram, and pulse oximetry. After baseline MRI and MAP measurements, the norepinephrine infusion rate was increased every 2 min by 0.01 to 0.04 μg · kg^−1^ · min^−1^ to achieve a 20% MAP increase or until a 200-mmHg systolic blood pressure limit. MRI scans were repeated. The process lasted about 30 to 40 min. All participants were monitored by an anesthesiologist, and norepinephrine infusion was stopped at any sign of adverse response. Three procedures were interrupted due to adverse events (ventricular bigeminy, claustrophobia, and vagal reaction), and two participants were excluded for not reaching target blood pressure or drug dose.

### Phase-contrast MRI

Details of the procedure are presented by Birnefeld *et al.*^8^ Region of interest for the internal/external carotid and vertebral arteries were manually drawn and kept constant throughout the cardiac cycle. Aortic planes were segmented semiautomatically, except in one case in which manual segmentation was required.^19^ Flow was assessed by integrating velocity within vessel-circumference region of interest. For assessment of CBF, a first PCMRI plane was placed at the C2–C3 level (retrospective gating using peripheral pulse recording; heart phases, 32; voxel size, 1 × 1 mm; 3-mm slice thickness; repetition time/echo time, 9.2 ms/5.5 ms; flip angle, 10°; velocity encoding, 80 cm/s). For assessment of cardiac output, a second plane was aligned perpendicularly to the ascending aorta, transecting the descending aorta (retrospective gating; heart phases, 32; voxel size, 2.5 × 2.5 mm; 8-mm slice thickness; repetition time/echo time, 4.2 ms/2.6 ms; flip angle, 10°; velocity encoding, 150 cm/s). Flow areas were calculated using region of interest for all vessels.

### Hemodynamic Characteristics

Total CBF was calculated as the sum of flow rates in the left and right internal carotid and vertebral arteries^14^; cardiac output represented ascending aorta flow rate; stroke volume was derived from cardiac output divided by heart rate; the external carotid arteries flows were summed and interpreted as a characterization of peripheral vascular flow. CVR was calculated as (MAP − intracranial pressure)/CBF. Systemic vascular resistance was calculated as ([MAP − central venous pressure]/cardiac output) × 80. Central venous pressure and intracranial pressure were both assumed to be 10 mmHg.^20,21^ To compare with transcranial Doppler, we estimated an equivalent flow velocity from PCMRI waveforms. This was done by extracting the peak systolic velocity and end-diastolic velocity from the flow waveform (internal/external carotid and vertebral arteries) and calculating a mean velocity analogously to the middle cerebral artery blood velocity index: [peak systolic velocity + (2 × end-diastolic velocity)]/3.^22^ No direct transcranial Doppler measurements were performed in this study.

### Compliance Estimates

#### Arterial Pulsatile Volume Load

Quantifying pulsatile blood flow measurements and PP can be used to determine the total cardiovascular compliance of vessels downstream from the measurement point. Specifically, compliance is assessed as the ratio of the pulsatile blood flow that contributes to accumulated pulsatile volume load (ΔV) to the PP. Two models are commonly used to estimate vascular compliance. The first model, ΔV/PP (compliance calculated as the ratio of pulsatile volume to pressure, C_VP_), assumes complete pressure dampening, leading to nonpulsatile capillary flow, where all measured pulsatile flow is attributed to arterial wall expansion. In contrast, the second model, the Windkessel approach (compliance estimated using the Windkessel model, C_WK_), assumes no pressure dampening before the capillaries, leading to pulsatile capillary flow proportional to PP. In this model, pulsatile capillary flow does not contribute to arterial expansion, producing a lower estimation of vascular compliance. These two approaches represent the extremes of physiologic assumptions. In this article, we estimate compliance for both systemic and cerebral vasculature using both models. By incorporating both approaches, we account for the full range of possibilities, whether fully damped or fully transmitted pressure pulsations, recognizing that the physiologic reality lies somewhere in between.

#### Flow

The mean flow rate (Q_mean_) and the systolic (Q_syst_) and the diastolic (Q_dias_) peaks of the measured flow waveforms (Q[t]) were determined. The peak-to-peak pulsatility amplitude, ΔQ, was calculated as the difference between the systolic and diastolic flow rates, expressed as ΔQ = Q_syst_ − Q_dias_.

#### Compliance Calculated as the Ratio of Pulsatile Volume to Pressure

For the first compliance model, we determined ΔV in the arterial tree distal to the measurement point. ΔV was calculated by subtracting Q_mean_ from Q(t), and integrating this difference from 0 to each time point t within one cardiac cycle of duration *t*_1_ to yield


$$\begin{array}{l} V\left(t\right)=\overset{t}{\underset{0}{\int }}\left(Q\left(\tau \right)-Q_{mean}\right)d\tau \end{array}$$ (eq. 1)

where V(t) is the volume function over the interval t ∈ [0, t_1_], and τ is the integration variable (time).

The ΔV was then calculated as


$$\begin{array}{l} \Delta V=\max\left(V\left(t\right)\right)-\min\left(V\left(t\right)\right) \end{array}$$ (eq. 2)

Now compliance downstream of the measurement point could be determined by the ratio of pulsatile volume load to pressure (C_VP_):


$$\begin{array}{l} C_{VP}=\frac{\;\Delta \;V}{PP} \end{array}$$ (eq. 3)

#### Windkessel Model (C_WK_)

**Fig. 2.**
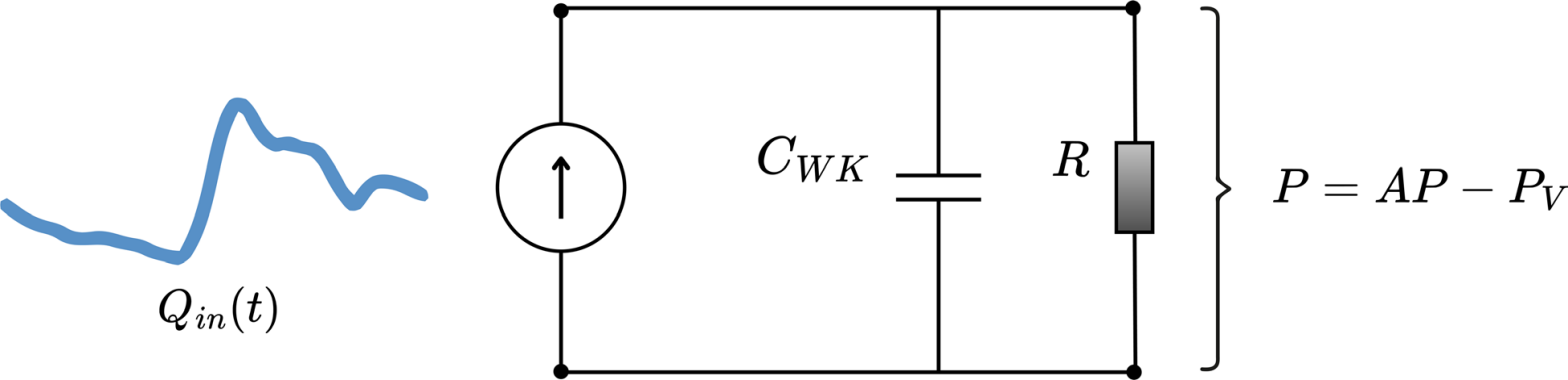
A schematic representation of the two-element Windkessel model illustrates a system driven by a measured flow, Q_in_(t), modeled as a current source. The flow encounters a parallel circuit that consists of a resistor, R, representing blood vessel flow resistance, and a capacitor, C_WK_, representing vascular compliance. The pressure drop, P, corresponds to the difference in pressure between the system’s entry and exit points. AP represents arterial pressure, while P_V_ corresponds to intracranial pressure in cerebrovascular system analysis and central venous pressure in systemic circulation analysis.


For the second model, the two-element Windkessel approach, everything downstream of the measurement point was modeled by one lumped resistance, *R*, in parallel with a lumped compliance/capacitance, C_WK_ (fig. 2). The resistance was calculated from

$$\begin{array}{l} R=\frac{MAP-P_{v}}{Q_{mean}} \end{array}$$ (eq. 4)

where P_v_ is the pressure reference (intracranial pressure for the cerebrovascular system and central venous pressure otherwise). The compliance for the Windkessel model, C_WK_, was found by solving the differential equation for the parallel circuit, using the pulsatile flow rate curve $Q_{in}\left(t\right)=Q\left(t\right)-Q_{mean}$ as input. Different values for C_WK_ were tested, finding the value that correctly yielded the measured PP—*i.e.*, C_WK_ was optimized to find the best fit for the measured PP. The differential equation was derived from circuit theory, where the flow into the circuit must equal the flow out


$$\begin{array}{l} Q_{in}\left(t\right)=Q_{R}\left(t\right)+Q_{C}\left(t\right) \end{array}$$ (eq. 5)

where $Q_{R}\left(t\right)$ and $Q_{C}\left(t\right)$ are the flow to the resistor and capacitor, respectively. Since


$$\begin{array}{l} Q_{R}\left(t\right)=P\left(t\right)/R \end{array}$$ (eq. 6)

where P(t) is the pressure across the resistor (and the capacitor), and flow through a capacitor is defined by


$$\begin{array}{l} C_{WK}\frac{dP\left(t\right)}{dt}=Q_{C}\left(t\right), \end{array}$$ (eq. 7)

the differential equation to solve becomes


$$\begin{array}{l} C_{WK}\frac{dP\left(t\right)}{dt}=Q_{in}\left(t\right)-\frac{P\left(t\right)}{R}. \end{array}$$ (eq. 8)

For a given value of the compliance parameter C_WK_, and with the resistance *R* calculated according to equation 4, the measured flow rate curves $Q_{in}\left(t\right)$ are used as input to equation 8 to solve for the pressure function P(t), representing the pulsatile blood pressure over time. The PP is then calculated as the difference between the maximum and minimum values of P(t). By comparing this calculated PP to the measured PP, the value of C_WK_ that best matches the observed data can be identified.

#### Pulsatility Index

Pulsatility, tied to arterial elasticity, reflects the role of large arteries as reservoirs during systole and diastole and is examined in various clinical applications.^23^ Arterial pulsations were analyzed using Gosling’s pulsatility index, calculated from flow waveforms as the amplitude divided by the mean flow.^24^


$$\begin{array}{l} Pulsatility\;Index=\frac{\;\Delta \;Q}{Q_{mean}} \end{array}$$ (eq. 9)

### Statistical Analysis

All analyses were performed using SPSS 29 (IBM Corp., USA). The optimization of C_WK_ was performed in Matlab (R2024b, MathWorks, USA) using the optimization function *fminsearch* and differential equation solver *ode23tb* applied to equation 8. The measured flow rates were interpolated using cubic splines before being fed into the model. The starting value for the pressure was set to 0 in the simulations (*i.e.*, P(0)=0)), and the value acquired from equation 3 was used as the initial guess for the compliance (“initial guess for C_WK_” = C_VP_). All tolerances were set to 10^−4^. Nonparametric tests were used throughout since the data set was small.^25^ Descriptive statistics were expressed as the median (interquartile range) if not otherwise specified. Changes in flow, pressure, resistance, compliance, and area variables during intravenous norepinephrine administration were compared to baseline using the Wilcoxon signed-rank test. Observed changes by sex were compared using Mann–Whitney U tests, analyzing relative changes in CBF, cardiac output, C_WK_, C_VP_, and pulsatility index during norepinephrine administration. Spearman correlation coefficients were calculated between pressure and flow at baseline MAP, for relative changes, and at the elevated MAP level. Additionally, correlations were calculated between pulsatility index in CBF and CVR at each level and change. A two-sided *P* value < 0.05 was considered statistically significant.

## Results

The final study population included 18 subjects (9 women) with a median age of 34 yr (32 yr to 38 yr), body weight of 72 kg (64 kg to 89 kg), and height of 174 cm (168 cm to 183 cm). CBF at higher MAP was analyzed in 17 subjects; 1 was excluded due to a nonperpendicular internal carotid artery. External carotid artery flow was not measurable in one subject due to anatomical constraints.

### Compliance and Pulsatility Index

**Fig. 3.**
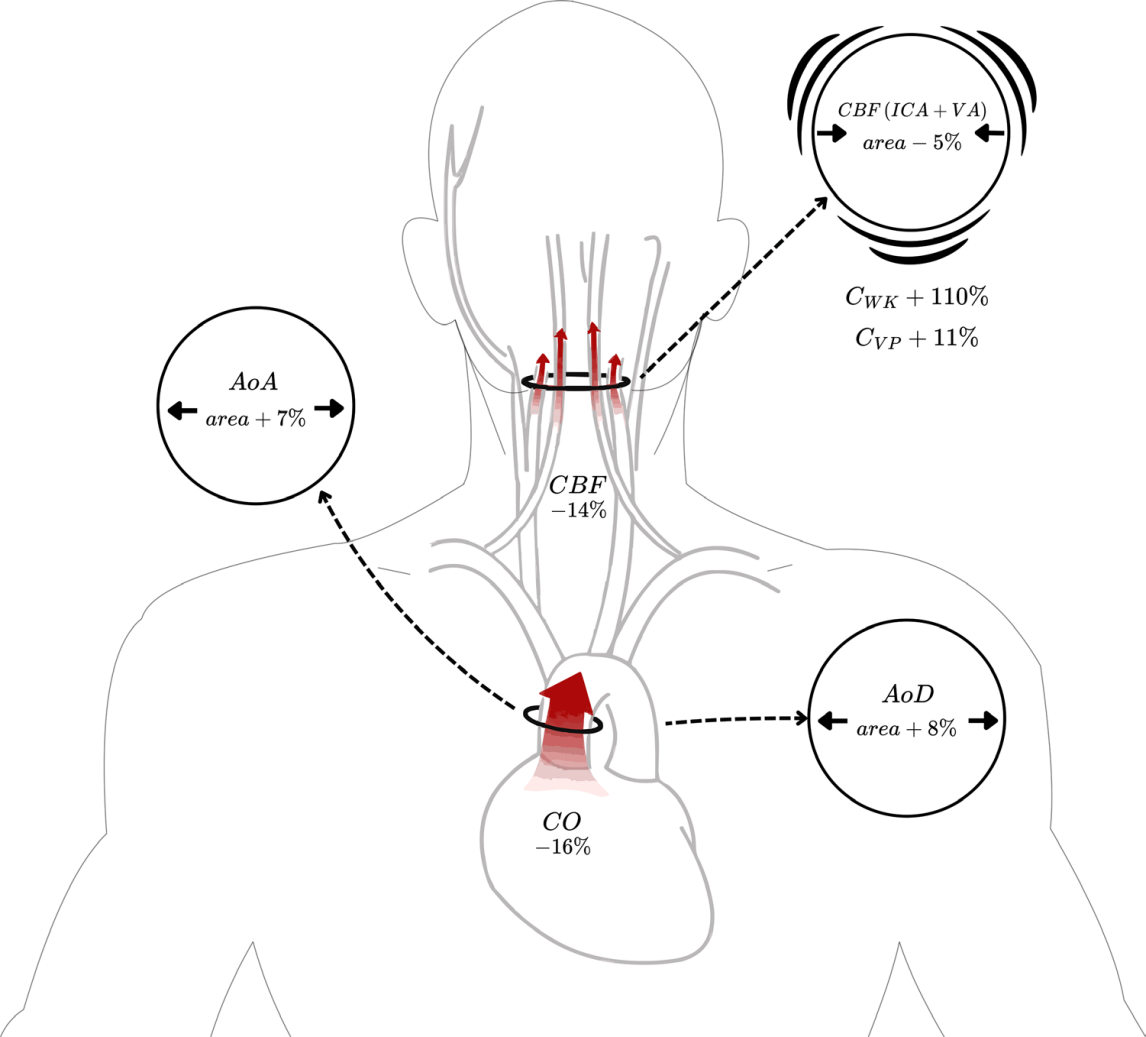
Diagram illustrating the changes in cardiovascular and cerebrovascular parameters during induced hypertension, MAP + 23%. AoA, ascending aorta; AoD, descending aorta; CBF, cerebral blood flow; CO, cardiac output; C_VP_, compliance—ratio of volume load to pressure change; C_WK_, compliance estimated using the Windkessel model; ICA, internal carotid artery; VA, vertebral artery.

Increasing blood pressure led to a median (interquartile range [IQR]) 110% increase in C_WK_ (56 to 163%; *P* = 0.001) and an 11% increase in C_VP_ of the cerebral arteries (−2 to 26%; *P* = 0.018; table 1; fig. 3). In absolute terms, the change in C_WK_ (0.01 ml/mmHg) was approximately threefold greater than the corresponding change in C_VP_ (0.003 ml/mmHg). Within the external carotid artery, C_WK_ increased by 12% (1 to 32%; *P* = 0.037), while there was no difference for C_VP_. There was no difference in systemic compliance in the ascending or descending aorta for C_WK_. C_VP_ decreased by 5% (−11 to 2%; *P* = 0.028) in the descending aorta, while no change in C_VP_ was observed in the ascending aorta. Pulsatility index increased by 18% in the ascending aorta (7 to 32%; *P* = 0.002) and 23% in the descending aorta (11 to 52%; *P* < 0.001) but remained unchanged for CBF and external carotid artery (table 1). No sex differences were observed in the relative changes of C_WK_/C_VP_/pulsatility index across flow measurements. No correlation was identified between pulsatility index in CBF and CVR at any level or change.

**Table 1.** Compliance Estimates and Pulsatility Index

Parameter	Baseline	Increased MAP	*P* Value
Windkessel compliance (ml/mmHg)			
C_WK_ CBF	0.008 (0.007–0.013)	0.018 (0.016–0.022)*	0.001
C_WK_ ECA	0.008 (0.006–0.014)*	0.009 (0.008–0.010)*	0.037
C_WK_ AoA	0.87 (0.70–1.00)	0.79 (0.71–0.98)	0.913
C_WK_ AoD	0.57 (0.50–0.69)	0.58 (0.46–0.60)	0.102
C_VP_, ml/mmHg			
C_VP_ CBF	0.025 (0.021–0.029)	0.028 (0.023–0.032)*	0.018
C_VP_ ECA	0.009 (0.007–0.017)*	0.010 (0.009–0.016)*	0.602
C_VP_ AoA	0.89 (0.73–1.03)	0.82 (0.73–0.98)	0.557
C_VP_ AoD	0.59 (0.52–0.71)	0.59 (0.48–0.66)	0.028
Pulsatility index			
PI CBF	0.95 (0.88–1.09)	1.01 (0.90–1.13)*	0.287
CBF ΔQ, ml/s	12.63 (11.27–14.65)	11.47 (9.78–13.27)*	0.135
CBF Q_mean_, ml/s	12.90 (11.22–14.37)	11.76 (9.85–12.41)*	< 0.001
PI ECA	2.30 (1.93–2.58)*	2.03 (1.79–2.63)*	0.463
PI AoA	3.98 (3.69–4.54)	4.82 (4.23–5.34)	0.002
PI AoD	4.38 (4.00–4.81)	5.57 (4.81–6.60)	< 0.001

Median (interquartile range).

* n = 17.

AoA, ascending aorta; AoD, descending aorta; CBF, cerebral blood flow; C_VP_, compliance – ratio of volume load to pressure change; C_WK_, Windkessel compliance; ECA, external carotid artery; MAP, mean arterial pressure; PI, pulsatility index; ΔQ, peak-to-peak pulsatility amplitude; Q_mean_, mean flow rate.

### Flow Area

At higher MAP, the total CBF cross-sectional area decreased by a median (IQR) of 5% (−17 to −3%; *P* = 0.033; table 2; fig. 3). In contrast, the mean cross-sectional areas of the ascending and the descending aorta increased by 7% (4 to 11%; *P* = 0.012) and 8% (6 to 11%; *P* < 0.001), respectively. The area difference between systole and diastole (maximum area minus minimum area) also increased in both the ascending aorta by 22% (10 to 47%; *P* = 0.002) and the descending aorta by 6% (−5 to 36%; *P* = 0.047). No significant changes were observed in the external carotid artery.

**Table 2.** Arterial Geometry

Flow Area	Baseline	Increased MAP	*P* Value
CBF mean, cm^2^	0.914 (0.800–1.002)	0.835 (0.721–0.932)*	0.033
ECA mean, cm^2^	0.316 (0.259–0.457)*	0.325 (0.220–0.391)*	0.435
AoA mean, cm^2^	7.002 (5.692–8.711)	7.534 (6.322–8.844)	0.012
AoA Δa	1.147 (1.009–1.469)	1.332 (1.133–1.778)*	0.002
AoD mean, cm^2^	4.284 (3.559–4.762)	4.537 (3.782–5.170)	< 0.001
AoD Δa	0.714 (0.587–0.886)	0.817 (0.683–0.989)	0.047

Median (interquartile range).

* n = 17.

Δa, difference between maximal and minimum flow area through cardiac cycle; AoA, ascending aorta; AoD, descending aorta; CBF, cerebral blood flow; ECA, external carotid artery.

### Flow and Pressures

The hemodynamic characteristics at baseline and increased MAP are presented in table 3 and depicted in figure 3. During the norepinephrine infusion at a median (IQR) rate of 0.17 µg · kg⁻^1^ · min⁻^1^ (0.14 µg · kg⁻^1^ · min⁻^1^ to 0.22 µg · kg⁻^1^ · min⁻^1^), MAP increased by 23% (19 to 29%; *P* < 0.001), while CBF decreased by 14% (−18% to −10%; *P* < 0.001), CVR increased by 49% (38 to 59%; *P* < 0.001), systemic vascular resistance increased by 60% (21 to 66%; *P* < 0.001), and cardiac output decreased by 16% (−23% to −15%; *P* = 0.011). Men showed a larger relative reduction in CBF in response to norepinephrine compared to women (−18% [−19 to −14%] *vs*. −10% [−14 to −4%]; *P* = 0.036), while no sex difference was observed for cardiac output or external carotid arteries. No correlations were identified between MAP and CBF/cardiac output/external carotid arteries at any level, and no significant differences in peak or mean velocities were observed. Additional descriptive statistics, including mean (95% CI), are presented in Supplemental Digital Content tables S1 to S6 (https://links.lww.com/ALN/E125) to complement the median (IQR) values reported in the main text.

**Table 3.** Hemodynamic Characteristics at Baseline and after Increased Blood Pressure

Parameter	Baseline	Increased MAP	*P* Value
Flow			
CBF, ml/min	774 (673 to 862)	705 (591 to 744)*	< 0.001
ECA, ml/min	182 (140 to 273)*	166 (128 to 219)*	0.015
CO, ml/min	5,874 (5,199 to 6,355)	4,995 (4,705 to 5,635)	0.011
SV, ml	93 (81 to 100)	100 (88 to 112)	0.002
HR, bpm	66 (56 to 74)	53 (46 to 62)	< 0.001
Velocity, cm/s			
ICA peak	50 (46 to 55)	49 (41 to 57)*	0.309
ICA mean	8 (6 to 11)	9 (−7 to 12)*	0.831
VA peak	34 (31 to 41)	34 (30 to 39)	0.053
VA mean	8 (6 to 9)	8 (6 to 11)	0.306
ECA peak	50 (40 to 58)*	45 (35 to 51)*	0.055
ECA mean	11 (6 to 12)*	8 (5 to 11)*	0.084
AoA peak	97 (85 to 112)	93 (83 to 112)	0.528
AoA mean	9 (−4 to 18)	4 (−17 to 14)	0.327
Pressure, mmHg			
MAP	87 (80 to 92)	107 (98 to 110)	< 0.001
SAP	130 (122 to 139)	156 (144 to 163)	< 0.001
DAP	65 (59 to 72)	81 (73 to 83)	< 0.001
PP	58 (52 to 63)	66 (62 to 70)	< 0.001
Resistance			
SVR, dyn · s · cm^−5^	1,053 (842 to 1,187)	1,500 (1,288 to 1,690)	< 0.001
CVR, mmHg · ml^−1^ · min^−1^	0.091 (0.083 to 0.115)	0.131 (0.125 to 0.164)*	< 0.001

Median (interquartile range).

* n = 17.

AoA, ascending aorta; CBF, cerebral blood flow; CO, cardiac output; CVR, cerebrovascular resistance; DAP, diastolic arterial pressure; ECA, external carotid artery; HR, heart rate; ICA, internal carotid artery; MAP, mean arterial pressure; PP, pulse pressure; SAP, systolic arterial pressure; SV, stroke volume; SVR, systemic vascular resistance; VA, vertebral artery.

## Discussion

This study highlights the role of compliant cerebral arteries in reducing fluctuations in pressure and flow pulsatility during induced hypertension, providing a more comprehensive description of the relationship between blood pressure and CBF. In healthy and awake individuals, regardless of whether pressure pulsatility was assumed to be fully attenuated before the capillary bed or completely transmitted to it, representing two extreme scenarios, we observed an increase in cerebrovascular compliance in response to increased blood pressure.

Understanding the assumptions of damped pulsatility is crucial for interpreting these results. If we assume that all pulsatility is dampened at the capillary level, then C_VP_ becomes the key measure to examine. Under this assumption, the results suggest that cerebrovascular compliance increased, while systemic vascular compliance from the descending aorta decreased. No change was observed in compliance from the ascending aorta, but it is important to note that this measurement includes cerebrovascular compliance as part of the calculation. On the other hand, if we assume that all pressure pulsatility is retained at the capillary level, then C_WK_ becomes the measure to examine. In this case, both cerebrovascular compliance and external carotid artery compliance increased, while no change was observed in the systemic circulation. Now, it is commonly believed that the pulsatile flow generated by the heart is fully dampened by the time it reaches the microcirculation. However, as human physiology operates along a continuum rather than at fixed extremes, some degree of pulsatility likely persists, varying in magnitude across different regions of the body.^26,27^ Despite representing extreme assumptions, both models agree that cerebrovascular compliance increases during norepinephrine-induced hypertension, while systemic compliance remains unchanged, providing compelling support for this as a real physiologic effect in the cerebral system.

Cerebrovascular compliance has previously been reported to increase during both induced hypertension and hypotension.^6,9^ Although seemingly contradictory, this can be explained by two physiologic mechanisms. In the case of induced hypertension, an increase in compliance requires that the increase in pulsatile volume exceeds the increase in blood pressure, as was the case in our study. In contrast, during induced hypotension, an apparent increase in compliance may be inferred if the reduction in blood pressure is greater than the decrease in pulsatile volume or in blood velocity, which is often used as a surrogate measure.^6,9^ During the pharmacologically induced hypertension, vasoconstriction occurred, as evidenced by the increased CVR and reduced cross-sectional CBF area.^28^ An important distinction is that the cross-sectional flow area remained unchanged in the external carotid arteries or even increased in the systemic circulation, where no increase in compliance was observed. A possible physiologic explanation is that this vasoconstriction positions the cerebral arterial walls at a more optimal point on the stress–strain curve, potentially enhancing their ability to accommodate increased pressure.^29–31^ This adjustment optimizes the vessel wall’s elasticity, similar to a rubber band that is partially stretched rather than fully extended, allowing it to absorb additional tension, dampen rapid pressure fluctuations, and protect downstream microcirculation from excessive pulsatility.^32–34^ While this concept is supported by general vascular biomechanics and experimental models of compliance regulation, direct evidence in humans remains scarce.^35^ Whether this represents a previously underappreciated element of cerebral autoregulation warrants further investigation.

Although CVR increased, pulsatility index remained unchanged, an observation that warrants further analysis. Pulsatility index, often used as a surrogate for CVR, is defined as the ratio of pulsatile (ΔQ) to mean CBF (Q_mean_), with the pulsatile component influenced by PP, particularly in the presence of arterial stiffening and small vessel disease.^15^ Under conditions of elevated CVR, reduced CBF, and increased PP, an increase in pulsatility index would typically be expected. A plausible explanation is preserved or even increased vascular compliance within the internal carotid and vertebral arteries, in line with our findings.^36^ Despite an increase in PP, ΔQ remained stable, indicating that these vessels continued to buffer pulsatile flow. This interpretation is further supported by the observed increase in pulsatility index in the ascending and descending aorta, where no change in compliance was detected.

This may also highlight the limitations of PCMRI as a tool for measuring peak velocities compared to transcranial Doppler. Peak velocity in a parabolic flow profile tends to be underestimated due to voxel and cardiac cycle averaging.^16^ Unlike MRI, transcranial Doppler offers superior temporal resolution, making it better suited for capturing rapid physiologic responses—a capability MRI has yet to achieve.^37^ Measured by transcranial Doppler, internal carotid artery systolic velocities in healthy individuals of the same age group are typically reported at approximately 80 cm/s.^38^ In contrast, our data demonstrated peak systolic velocities of about 50 cm/s both at baseline and under elevated blood pressure.

### Cerebral Blood Flow

Our results show that induced hypertension, characterized by a moderate increase in MAP, led to a considerable reduction in CBF and a substantial increase in CVR. These findings question the clinical value of induced hypertension as a therapeutic strategy. Additionally, cardiac output declined markedly, while systemic vascular resistance increased substantially; we found no correlation between the decrease in cardiac output and CBF, nor between changes in CVR and systemic vascular resistance, indicating that changes in cardiac output do not directly influence CBF in this context, supporting the expected view that blood flow control is different for the cerebral system compared to the systemic. While these findings suggest that elevating blood pressure may not effectively enhance CBF in healthy individuals, the response may differ in patients with cerebral pathology such as vasospasm, traumatic brain injury, or hepatic encephalopathy, where autoregulatory mechanisms are often impaired. Our finding that MAP increased while cardiac output decreased aligns with recent studies showing minimal correlation between these measures, especially in perioperative and critical care.^39,40^ This dissociation underscores the need for caution when interpreting MAP as a surrogate for systemic flow or perfusion, as elevated pressure may not necessarily reflect improved circulatory delivery. This has broader implications for hemodynamic management strategies where MAP and cardiac output are still often viewed interchangeably.

Reviews have examined the effects of norepinephrine on cerebrovascular response and CBF.^41,42^ Studies conducted in healthy adults, comparable to ours, consistently reported that middle cerebral artery blood velocity, used as a surrogate for CBF, remained stable when measured with transcranial Doppler. In contrast, our study demonstrated a reduction in CBF. The discrepancy may result because transcranial Doppler measures blood flow velocity in intracranial arteries instead of volumetric flow, accurately reflecting CBF only if the vessel diameter remains constant.^43^ In our study, the significant reductions in the summed flow areas of the internal carotid and vertebral arteries decreased CBF. But importantly, no significant difference would have been observed if we had analyzed peak or mean velocities (cm/s) instead of flow (ml/min).

The decrease in CBF was more pronounced in men than women, despite similar reductions in cardiac output between sexes. This sex difference suggests possible structural or functional differences in cerebrovascular reactivity to alterations in blood pressure.^44^ Nonetheless, literature on sex-specific effects on CBF in response to norepinephrine-induced blood pressure elevation remains limited.^41,42^ These findings underline the need to consider and further investigate sex as a potential modifier in vascular response studies, which may help inform sex-specific strategies for managing hypertensive effects on cerebral circulation.

### Limitations

While this study highlights the role of compliant arteries in mitigating hypertension-induced flow fluctuations, there are some limitations that should be noted. The small sample size and focus on young, healthy individuals may limit generalizability to older or cardiovascular-compromised populations. PP, used in compliance calculations, was not measured in the cerebral arteries, although correction factors were applied for carotid-to-radial amplification. Moreover, the PP in the aorta is likely even lower, but this is of little significance as long as pressure changes remain correlated.^45,46^ Additionally, uncontrolled arterial partial pressure of carbon dioxide (Paco₂) levels, which regulate CBF, could have influenced results, as norepinephrine-induced reductions in Paco₂ can lead to cerebral vasoconstriction and changes in arterial diameters.^47–49^ Furthermore, caution must be exercised in extrapolating these results to patients with intracranial pathologies. Conditions such as traumatic brain injury, subarachnoid hemorrhage, or space-occupying lesions may impair cerebral autoregulation, intracranial pressure regulation, and vascular compliance.^50,51^ In such populations, the cerebrovascular response to increased blood pressure could differ markedly from that observed in our study. Therefore, further studies are warranted to investigate these mechanisms in clinical contexts where induced hypertension is commonly employed.

### Conclusions

This study examined the physiologic response of cerebral circulation to induced hypertension using PCMRI in awake and healthy individuals. Compliance increased in cerebral arteries during induced hypertension, regardless of whether PP was assumed to be fully attenuated before or completely transmitted through the capillary bed. This response represents an additional factor complicating the interplay between blood pressure and CBF, illustrating that, while increased pressure by norepinephrine surprisingly leads to decreased CBF, a protective function against heightened pulsatile flow appears preserved through increased vascular compliance. Future research could explore its significance across different disease states or contexts. Moreover, despite technical challenges, PCMRI may in the future aid in evaluating cerebral perfusion and vascular compliance in critically ill patients, many of whom already undergo MRI as part of standard care.

### Acknowledgments

The authors acknowledge J. Plese, M.Sc. (Department of Anesthesiology and Intensive Care, Umeå University Hospital, Umeå, Sweden), for her valuable assistance with participant recruitment and overseeing the study procedures. They also extend their thanks to R. de Peredo-Axelsson, B.Sc., and H. Israelsson, B.Sc. (Department of Radiology, Umeå University Hospital), as well as J. Hauksson, Ph.D. (Department of Radiation Sciences, Umeå University Hospital), for their contributions to MRI data acquisition. Special thanks are given to Björn Pilebro, M.D., Ph.D. (Department of Public Health and Clinical Medicine, Umeå University Hospital), for his guidance on risk assessments, and to Davide Vanoli, M.D., Ph.D. (Department of Public Health and Clinical Medicine, Umeå University Hospital), for his expertise and support in analyzing the pulsatility index.

### Research Support

This research was supported by Umeå University (Umeå, Sweden), Region Västerbotten (Umeå, Sweden), the Swedish Heart-Lung Foundation (Stockholm, Sweden, grant No. 20220397 to Dr. Zarrinkoob), and the Swedish Research Council (Stockholm, Sweden, grant No. VR-2021-00711 to Prof. Eklund).

### Competing Interests

The authors declare no competing interests.

## Supplemental Digital Content

Supplemental Tables S1 to S6, https://links.lww.com/ALN/E125

## Supplementary Material
