# Supplementary material for: Cerebral Blood Flow under Pressure: Investigating Cerebrovascular Compliance with Phase-contrast Magnetic Resonance Imaging during Induced Hypertension
Source: Anesthesiology. 2025 Jul 11;143(4):917–28. doi: 10.1097/ALN.0000000000005651 (PMC12416895; doi:10.1097/ALN.0000000000005651)
Supplement: Supplementary file 1 [file aln-143-917-s001.pdf]

	Baseline	Increased MAP	*P*
**Windkessel Compliance (ml/mmHg)**			
C_{WK} CBF	0.009 (0.007 to 0.012)	0.019 (0.015 to 0.021)*	**0.001**
C_{WK} ECA	0.009 (0.007 to 0.012)*	0.011 (0.008 to 0.013)*	**0.037**
C_{WK} AoA	0.84 (0.75 to 0.93)	0.84 (0.75 to 0.93)	**0.913**
C_{WK} AoD	0.59 (0.52 to 0.65)	0.56 (0.51 to 0.62)	**0.102**
**C_{VP} (ml/mmHg)**			
C_{VP} CBF	0.025 (0.023 to 0.028)	0.028 (0.025 to 0.030)*	**0.018**
C_{VP} ECA	0.011 (0.009 to 0.014)*	0.012 (0.009 to 0.014)*	**0.602**
C_{VP} AoA	0.88 (0.77 to 0.97)	0.86 (0.78 to 0.95)	**0.557**
C_{VP} AoD	0.62 (0.55 to 0.68)	0.58 (0.53 to 0.64)	**0.028**
**Pulsatility Index**			
PI CBF	0.98 (0.90 to 1.06)	1.03 (0.96 to 1.11)*	**0.287**
CBF ΔQ (ml/s)	12.86 (11.68 to 14.04)	12.75 (10.56 to 12.94)*	**0.135**
CBF Q_{mean} (ml/s)	13.24 (12.01 to 14.47)	11.75 (10.56 to 12.94)*	**<0.001**
PI ECA	2.30 (2.07 to 2.54)*	2.15 (1.95 to 2.34)*	**0.463**
PI AoA	4.09 (3.78 to 4.40)	4.76 (4.34 to 5.17)	**0.002**
PI AoD	4.36 (4.03 to 4.70)	5.57 (5.04 to 6.11)	**<0.001**

*Table S1 Compliance estimates and pulsatility index. Mean (95% CI). C_{WK} indicates Windkessel compliance; CBF, Cerebral Blood Flow; ECA, External Carotid Artery; AoA, Ascending Aorta; AoD, Descending Aorta; C_{VP}, compliance - ratio of volume load to pressure change; PI, Pulsatility Index, ΔQ, max-min flow; Q_{mean}, mean flow. *; n=17.*

	**Baseline**	**Increased MAP**	***P***
**Flow area**			
CBF mean, cm^2	0.900 (0.835 to 0.965)	0.823 (0.747 to 0.899)*	**0.033**
ECA mean, cm^2	0.347 (0.286 to 0.408)*	0.332 (0.270 to 0.393)*	**0.435**
AoA mean, cm^2	7.330 (6.364 to 8.296)	7.723 (6.786 to 8.659)	**0.012**
AoA Δa	1.196 (1.000 to 1.392)	1.533 (1.207 to 1.859)*	**0.002**
AoD mean, cm^2	4.155 (3.776 to 4.535)	4.491 (4.079 to 4.902)	<**0.001**
AoD Δa	0.735 (0.648 to 0.822)	0.845 (0.734 to 0.957)	**0.047**

*Table S2 Arterial geometry. Mean (95% CI). CBF, Cerebral Blood Flow; ECA, External Carotid Artery; AoA, Ascending Aorta; and AoD, Descending Aorta; Δa, max-min area through cardiac cycle. *; n=17.*

	Baseline	Increased MAP	*P*
**Flow**			
CBF (ml/min)	795 (721 to 868)	687 (621 to 753)*	**<0.001**
ECA (ml/min)	199 (161 to 237) *	171 (147 to 196)*	**0.015**
CO (ml/min)	6011 (5461 to 6560)	5313 (4837 to 5788)	**0.011**
SV (ml)	93 (86 to 99)	101 (92 to 109)	**0.002**
HR (bpm)	66 (60 to 71)	53 (49 to 58)	**<0.001**
**Velocity (cm/s)**			
ICA Peak	51 (48 to 55)	50 (45 to 55)*	**0.309**
ICA Mean	9 (7 to 11)	6 (1 to 11) *	**0.831**
VA Peak	38 (34 to 42)	36 (32 to 40)	**0.053**
VA Mean	8 (5 to 10)	7 (5 to 10)	**0.306**
ECA Peak	50 (43 to 57) *	44 (38 to 50) *	**0.055**
ECA Mean	9 (5 to 13) *	6 (2 to 11)*	**0.084**
AoA Peak	100 (90 to 110)	98 (88 to 108)	**0.528**
AoA Mean	3 (-8 to 14)	-1 (-13 to 10)	**0.327**
**Pressure (mmHg)**			
MAP	85 (80 to 89)	105 (101 to 109)	**<0.001**
SAP	131 (125 to 136)	154 (148 to 160)	**<0.001**
DAP	64 (59 to 68)	78 (74 to 82)	**<0.001**
PP	58 (54 to 62)	65 (61 to 69)	**<0.001**
**Resistance**			
SVR ($dyn \cdot secs \cdot cm^{-5}$)	1021 (915 to 1126)	1465 (1330 to 1599)	**<0.001**
CVR ($mmHg \cdot ml^{-1} \cdot min^{-1}$)	0.096 (0.086 to 0.107)	0.142 (0.127 to 0.157)*	**<0.001**

Table S3 Hemodynamic characteristics at baseline and after increased blood pressure. Mean (95% CI). CBF, Cerebral Blood Flow; ECA, External Carotid Artery; CO, Cardiac Output; SV, Stroke Volume; HR, Heart Rate; ICA, Internal carotid artery; VA, Vertebral artery; AoA, Ascending Aorta; MAP, Mean Arterial Pressure; SAP, Systolic Arterial Pressure; DAP, Diastolic Arterial Pressure; PP, Pulse Pressure; SVR, Systemic Vascular Resistance; CVR, Cerebral Vascular Resistance.

**; n=17*

	**Relative change (%) at increased MAP, mean (95% CI)**	***P***
**Windkessel Compliance**		
C_{WK} CBF	112 (74 to 150) †	**0.001**
C_{WK} ECA	21 (1 to 40)*	**0.037**
C_{WK} AoA	0 (-4 to 4)	**0.913**
C_{WK} AoD	-3 (-8 to 1)	**0.102**
**C_{VP} (ml/mmHg)**		
C_{VP} CBF	12 (3 to 21)*	**0.018**
C_{VP} ECA	8 (-5 to 20)*	**0.602**
C_{VP} AoA	-1 (-5 to 3)	**0.557**
C_{VP} AoD	-5 (-9 to 0)	**0.028**
**Pulsatility Index**		
PI CBF	7 (-2 to 16)*	**0.287**
CBF ΔQ (ml/s)	-7 (-15 to 1)*	**0.135**
CBF Q_{mean} (ml/s)	-13 (-17 to -9)*	**<0.001**
PI ECA	-4 (-15 to 8)*	**0.463**
PI AoA	17 (9 to 26)	**0.002**
PI AoD	29 (17 to 41)	**<0.001**

*Table S4 Compliance estimates and pulsatility index, relative changes. Mean (95% CI). C_{WK} indicates Windkessel compliance; CBF, Cerebral Blood Flow; ECA, External Carotid Artery; AoA, Ascending Aorta ; AoD, Descending Aorta; C_{VP}, compliance - ratio of volume load to pressure change; PI, Pulsatility Index, ΔQ, max-min flow; Q_{mean}, mean flow, *; n=17, † ; n=16.*

	**Relative change (%) at increased MAP, mean (95% CI)**	***P***
**Flow area**		
CBF mean, cm^2	-7 (-13 to -1)*	**0.033**
ECA mean, cm^2	-1 (-19 to 18) †	**0.435**
AoA mean, cm^2	6 (3 to 9)	**0.012**
AoA Δa	33 (13 to 52)*	**0.002**
AoD mean, cm^2	8 (6 to 10)	**<0.001**
AoD Δa	18 (2 to 35)	**0.047**

*Table S5 Arterial geometry, relative changes. Mean (95% CI). CBF, Cerebral Blood Flow; ECA, External Carotid Artery; AoA, Ascending Aorta; and AoD, Descending Aorta; Δa, max-min area through cardiac cycle. *; n=17, † ; n=16.*

	**Relative change (%) at increased MAP, (95% CI)**	***P***
**Flow**		
CBF (ml/min)	-13 (-17 to -9)*	**<0.001**
ECA (ml/min)	-10 (-19 to 0)*	**0.015**
CO (ml/min)	-10 (-19 to -2)	**0.011**
SV (ml)	9 (4 to 14)	**0.002**
HR (bpm)	-18 (-24 to -12)	**<0.001**
**Pressure (mmHg)**		
MAP	24 (21 to 28)	**<0.001**
SAP	18 (16 to 20)	**<0.001**
DAP	24 (19 to 30)	**<0.001**
PP	13 (9 to 18)	**<0.001**
**Resistance**		
SVR ($dyn \cdot secs \cdot cm^{-5}$)	47 (34 to 60)	**<0.001**
CVR ($mmHg \cdot ml^{-1} \cdot min^{-1}$)	48 (40 to 56)*	**<0.001**

*Table S6 Hemodynamic characteristics at baseline and after increased blood pressure, relative changes. Mean (95% CI). CBF, Cerebral Blood Flow; ECA, External Carotid Artery; CO, Cardiac Output; SV, Stroke Volume; HR, Heart Rate; ICA, Internal carotid artery; VA, Vertebral artery; AoA, Ascending Aorta; MAP, Mean Arterial Pressure; SAP, Systolic Arterial Pressure; DAP, Diastolic Arterial Pressure; PP, Pulse Pressure; SVR, Systemic Vascular Resistance; CVR, Cerebral Vascular Resistance. *; n=17, † ; n=16.*
